# Deformable image registration in radiation therapy

**DOI:** 10.1002/jmrs.446

**Published:** 2020-10-26

**Authors:** Jason A Dowling, Laura M O’Connor

**Affiliations:** ^1^ CSIRO Australian E‐Health Research Centre Herston Queensland Australia; ^2^ School of Mathematical and Physical Sciences University of Newcastle Newcastle New South Wales Australia; ^3^ School of Information Technology and Electrical Engineering University of Queensland St Lucia Queensland Australia; ^4^ School of Information Technology and Systems University of Canberra Canberra Australian Capital Territory Australia; ^5^ Faculty of Medicine University of New South Wales Sydney New South Wales Australia; ^6^ Centre for Medical Radiation Physics University of Wollongong Wollongong New South Wales Australia; ^7^ Institute of Medical Physics University of Sydney Sydney New South Wales Australia; ^8^ Department of Radiation Oncology Calvary Mater Newcastle Hospital Newcastle New South Wales Australia; ^9^ School of Health Sciences University of Newcastle Newcastle New South Wales Australia

## Abstract

Deformable image registration is an increasingly important method to account for soft tissue deformation between image acquisitions. This editorial discusses the clinical need and current status of deformable image registration.

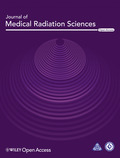

Radiation therapy has a history of innovation in applying computer technology to improve the accuracy and effectiveness of radiation treatment planning and delivery.^1^ Rigid image registration (RIR, involving rotation and translation) and fusion have been included in treatment planning software for years, and deformable image registration (DIR) is increasingly used to account for soft tissue deformation between image acquisitions. The American Association of Physicists in Medicine Radiation Therapy Committee Task Group No. 132 report^2^ provides an excellent overview of image registration methodology and quality assurance.

This edition of the journal includes two papers which focus on aspects of DIR in radiation therapy. The first is a report from the 2018 Deforming to Best Practice workshops held in Sydney and Melbourne. This paper by Barber et al.^3^ focuses on best practices and recommendations for the clinical use of commercial DIR tools. The second paper comprehensively reports on the use of the MIM Maestro (MIM Software Inc.) QA tool for evaluating DIR accuracy in head and neck cancer.^4^ Notably in addition to this tool, the MIM system also allows quantitative local deformation vector analysis through reporting of the Jacobian determinant (which indicates expansion, contraction and unnatural folding of registered tissues) and measures of propagated contour accuracy.

## Clinical Need for DIR

The clinical imaging information required for radiation therapy planning has increased with the continued uptake of advanced techniques such as volumetric modulated arc therapy (VMAT), stereotactic body radiation therapy (SBRT) and cranial stereotactic radiosurgery (CSRS). Due to limited soft tissue contrast in planning CT scans (pCT), MRI and PET can provide additional anatomical and functional information. Registration of this multi‐modality imaging to the planning CT scan increases the reliability and reproducibility of target region delineation in the contouring phase of radiation therapy planning.^5^, ^6^, ^7^, ^8^, ^9^

These multi‐modality image sets are registered using either RIR or DIR which can utilise image contrast, anatomical landmarks or fiducial markers as a registration reference. Rigid registration has been more commonly employed in the clinical setting and is provided in most planning and treatment platforms. It can be very useful between modalities with minimal anatomical changes. However, anatomy between modalities for a single patient (including tumour shape changes, weight changes, positional differences and internal variation) can indicate the need for DIR. DIR can also be very useful for MRI to pCT image registration, particularly as MR‐guided RT increases in popularity. In addition, DIR can be used for automated structure outlining and adaptive planning; and to assess how anatomical changes affect dose distribution, organ‐at‐risk dose levels and target coverage.

Daily treatment image matching has increased the use of cone beam CT (CBCT) to pCT matching over orthogonal KV to digitally reconstructed radiograph (DRR) matching. The image registration translation and sometimes rotational movements are then applied to the treatment couch, to ensure the treatment isocentre is located correctly within the patient.

## Common Clinical Problems with Registration

Image registration may introduce inaccuracies which can ultimately affect dose delivery to the target structures. The image registration process needs to be monitored to improve treatment reliability and accuracy. Common problems include:

### Patient Positioning and internal anatomy

Patient positioning for diagnostic MRI is usually determined by image quality, patient comfort and the positioning of coil arrays, while diagnostic PET imaging is focused on patient comfort, attenuation and improving imaging quality and visualisation. Patient positioning in radiation therapy usually uses ancillary immobilisation equipment with a focus on reproducibility and accessibility to the treatment region. As such, there are invariable acquisition discrepancies between these modalities. In head and neck imaging, the difference in neck flexion between diagnostic imaging and radiation therapy imaging can make the RIR process challenging, and translation of structures between the imaging modalities is difficult. Frequently in RIR, trade‐offs are made to best fit to the region of interest and then account for registration uncertainties in other parts of the dataset. Differences in patient positioning can make the registration unusable. DIR can assist with these issues to an extent; however, large variations in patient positioning and tissue volume can cause unrealistic results.

Rectal and bladder filling differences between pelvic image acquisitions can make accurate RIR difficult. Variations such as stomach contents or breathing period can also affect image registration for abdominal RIR. For liver SBRT, if the normal breathing amplitude is greater than approximately 1.5 cm, the breathing period of the scanning modality (inhalation or exhalation) can cause translational movement in addition to liver structure deformations. A solution is to scan the patient in the same breathing period for all modalities; however, this can be technically difficult. If this is not possible, secondary surrogate anatomical structures that are in the proximity of the tumour region, such as the hepatic ducts, can be used. Imaging with the patient in the treatment position, with the same internal anatomical conditions is the best way to improve both RIR and DIR results.

### Image distortion

Image distortion can cause registration issues, including insufficient target coverage. MRI is particularly susceptible to image distortion stemming from inhomogeneity of the static magnetic field, gradient non‐linearity, magnetic susceptibility of the patient and chemical shift. Minimising distortion in MRI often comes at a cost to image quality. In order to improve RIR when registering between a pCT and MRI, MRI protocols with decreased distortion should be used including the following: the use of high bandwidths; 1.5T over 3T; 3D distortion correction software; use of spin echo over gradient echo sequences; 3D‐based sequences over 2D, scanning near isocentre and the use of small field of views. Radiation therapy ancillary position equipment can make these distortion reduction goals difficult. As Barber et al. note,^3^ routine distortion QA is important (but is rarely performed on diagnostic MRIs).

Image registration algorithms generally operate in 3D, and 2D imaging, angle of slice plane, slice gaps and large slice thickness used in diagnostic imaging can impair RIR and DIR. When an MRI is fused to the pCT, these issues can reduce MRI quality or cause registration inaccuracies. Isotropic sequences scanned in treatment position can assist with registration results.

### Image artefacts

Image artefacts can affect registration, as artificially high or low intensity regions may be generated. Methods exist to reduce metal artefacts; however, the presence of metal prostheses in the treatment regions (such as bilateral hip prosthesis for prostate treatments) can render CBCT unusable due to restricted soft tissue information. Therefore, registration techniques on treatment are limited to oblique orthogonal kV imaging and the use of bony registration if fiducials are not present.

### Adaptive planning

Adaptive planning involves the use of DIR to register pCT Hounsfield Unit (HU) values to CBCT for dose planning purposes. DIR for adaptive planning is valuable, as it allows CBCTs acquired for treatment to be used to determine dose effects of daily set‐up variations, weight loss or rapid tumour response or progression. Reservations remain about the ability of DIR to correctly estimate CBCT HU values. Additionally, the insufficient field of view of CBCT cranio‐caudally and laterally (i.e. in the shoulder region) mean that many centres use this image to simply determine whether a CT rescan is required.

### Automatic segmentation of structures

DIR can be efficient for structure contouring, particularly with multi‐atlas approaches. Currently automatic contouring can work quite well for larger structures; however, for smaller structures or regions of variable anatomy, the structure deformation may be inaccurate.

## Summary

DIR is already effectively used clinically for adaptive planning and automatic contouring, as well as for some multi image registration corrections. DIR can be a useful clinical tool to further reduce errors in the RIR process. DIR algorithms may fail with bony contours (transforming or deforming the structures unnaturally) and can give unrealistic results, introducing new errors in the process. The success of DIR is also limited by the gross positional and anatomical difference between datasets. DIR could be of use for systematic errors such as image deformation in MRI but, as noted,^3^ commercially implemented DIR algorithms struggle to register modalities with different image contrast for a single structure.

The major sources of error in RIR and DIR stem from the positional and internal anatomical variations between diagnostic and radiation therapy imaging. Registration results could be improved through more effective communication. Increased collaboration with diagnostic departments could improve results from positioning (such as arms up vs arms down protocols), breath hold phase (inhalation or exhalation) and providing identical ancillary equipment.

There are several current research areas in DIR which are likely to feed into future commercial systems. To aid scientific reproducibility and knowledge sharing, many of these methods are available through open source repositories and toolkits (such as https://simpleitk.org/). These areas include structure guided, poly‐affine and discontinuous (to handle variations in organ motion, such as the lung) registration. There is also much interest and promising results in deep learning methods.^10^

In conclusion, there is a clinical need for DIR due to unavoidable anatomical differences resulting from different internal and positional conditions between modalities. Current commercially available DIR software needs greater accuracy before wide clinical acceptance. There is a need for greater understanding of how registration software is implemented and how to interpret QA tools, which will build greater trust in the output results. As demonstrated by the two papers in this issue, this process is currently underway.
